# Aplasia of the lacrimal and major salivary glands (ALSG). First case report in spanish population and review of the literature

**DOI:** 10.4317/jced.55350

**Published:** 2018-12-01

**Authors:** David Neagu, Beatriz Patiño-Seijas, Ramón Luaces-Rey, Javier Collado-López, Álvaro García-Rozado-González, José-Luis López-Cedrún-Cembranos

**Affiliations:** 1 MD. Maxillofacial Surgery Department, University Hospital A Coruña, Spain; 2 MD. Maxillofacial Surgery Department, Private Practice Pontevedra, Spain; 3 MD, DMD, PhD. Maxillofacial Surgery Department, University Hospital A Coruña, Spain; 4 MD, DDS, PhD. Head of Maxillofacial Surgery Department, University Hospital A Coruña, Spain

## Abstract

Aplasia of the lacrimal and the major salivary glands (ALSG) is a rare disorder with scarce cases described in the recent literature. The pattern of genetic inheritance is autosomal dominant with variable expressivity. A 40 years male patient was referred to the Oral and Maxillofacial Service at the Hospital Universitario de A Coruña diagnosed with complete agenesis of all salivary glands. Our case it is the first of ALSG syndrome in the Spanish literature. Imaging tests are necessary to confirm the lack of formation of salivary glands and alteration of lacrimal system. A mutation of FGF10 has been proposed as the responsible of the syndrome. The management of the lacrimal alteration depends of the clinical findings. Clinical suspicion remains the principal tool to diagnose the syndrome.

** Key words:**ALSG, salivary glands aplasia.

## Introduction

Aplasia of the lacrimal and the major salivary glands (ALSG) is a rare disorder with scarce cases described in the recent literature. The pattern of genetic inheritance is autosomal dominant with variable expressivity (1).

The salivary glands develop from the ectoderm, but a small part of submandibular and sublingual glands can grow from both ectoderm and endoderm (2). Their aplasia or hypoplasia of them is rare. Gruber was the first one who described a case of aplasia in 1885 (3).

Abnormal salivary glands can be associated with other ectodermal defects, such as the lack of development of the lacrimal apparatus, dental hipodontya, affectation of skin, hair and nails, constituting a syndrome known as lacrimo-auriculo-dento-digital (LADD) (4). Down syndrome, Treacher Collins syndrome, hemi-facial microstomia, cleft lip and palate, Klinefelter syndrome have also been associated with lack of formation of salivary glands (5-7).

## Case Report

A 40 years male patient was referred to the Oral and Maxillofacial Service at the Hospital Universitario de A Coruña diagnosed with complete agenesis of all salivary glands. He was Caucasian, his medical history revealed pollen allergy and asthma. He was an active smoker. No family history of genetic disease has been reported.

The patient was assessed by an internal medicine specialist with the suspicious of Sjörgen Syndrome. His dentist observed multiple caries associated with hyposalivation, despite a correct hygiene and regular dental revisions. A normal histological analysis of the minor salivary glands ruled out the possibility of Sjörgen Syndrome.

A blood test was performed with no significant alterations, but no genetic test of FGF10 was available in our center.

**Figure 1:**
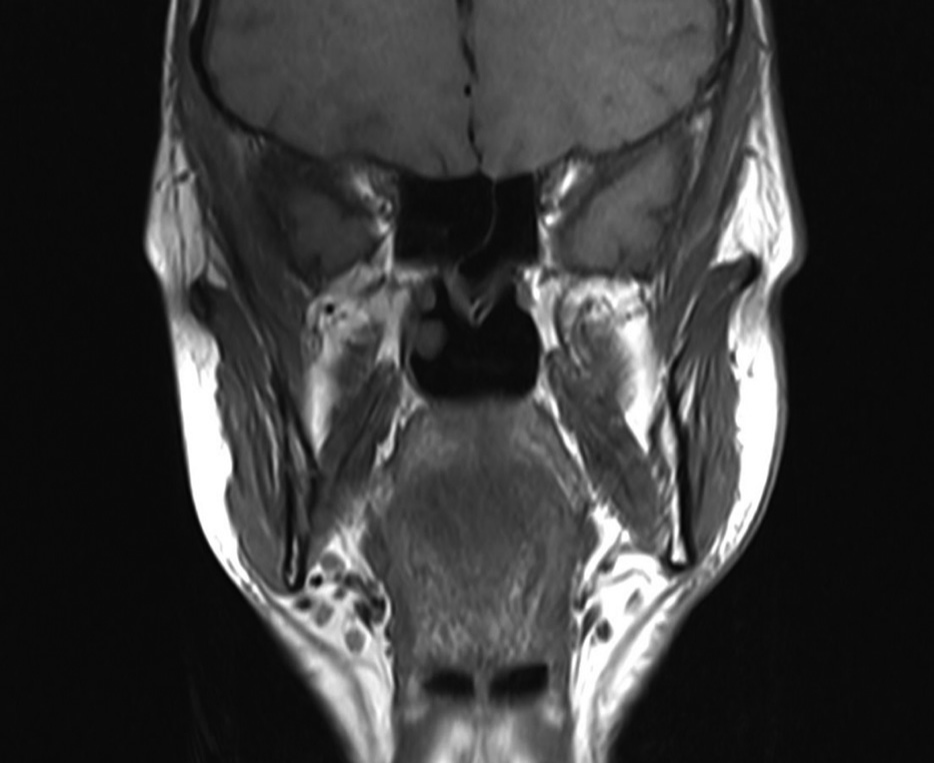
MRI revealing agenesis of all major salivary glands.

The initial salivary glands ultrasound suggested a hypoplasia or aplasia, MRI and Scintigraphy were performed and the aplasia of all major salivary glands was confirmed (Fig. 1).

**Figure 2:**
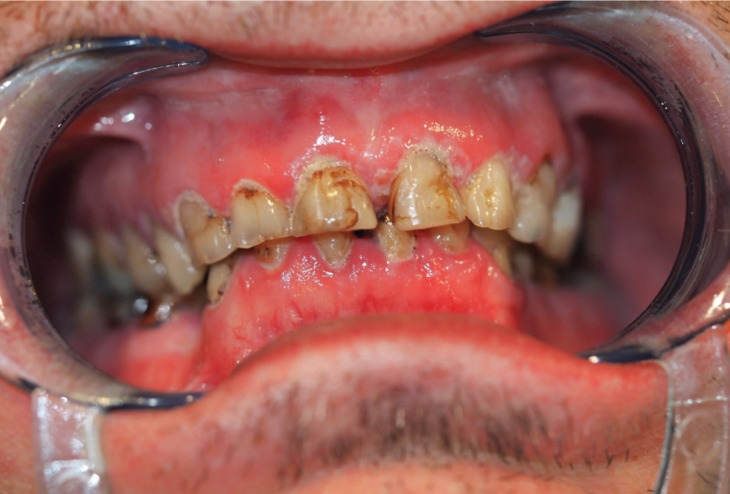
Multiple caries and dry mouth in physical exploration.

**Figure 3:**
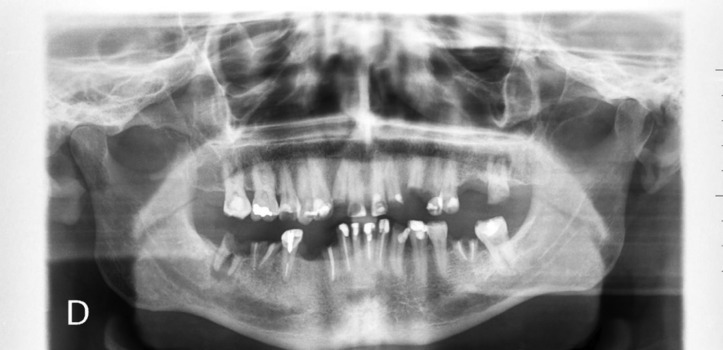
OPG revealing affectation of almost all dental pieces.

The physical examination in our service revealed numerous caries, dry mouth and the absence of various teeth, Stenon and Wharton ducts (Figs. 2,3).

**Figure 4:**
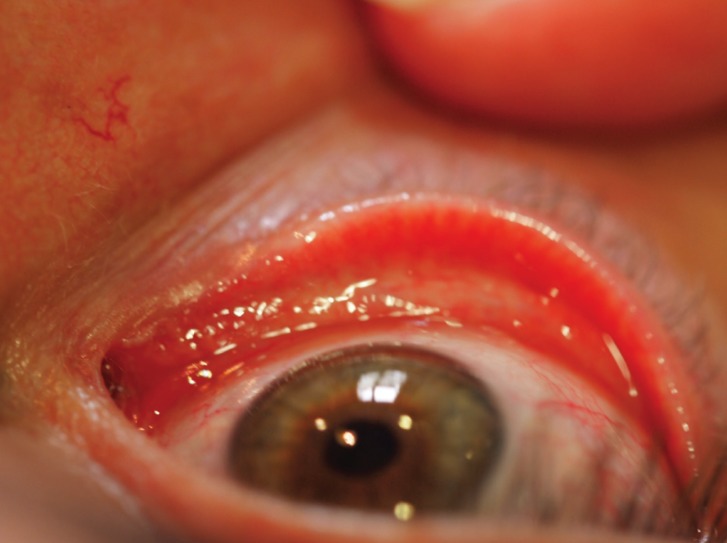
No tubes or lacrimal puncta are visualized in the ophthalmologic examination.

Regarding his ophthalmologic history, he required a surgical intervention and tube placement at the age of 10 because of lacrimal atresia of both eyes. A new placement of the tubes was necessary at age of 11. Nowadays, no tubes are visualized in the ophthalmologic examination (Fig. 4). The patient has neither dry eyes symptoms nor visual alterations. The Schirimer test shows 15mm in the right eye and 17mm in the left eye.

## Discussion

Aplasia or hypoplasia of the lacrimal and salivary glands (ALSG) is a rare syndrome, autosomal dominant with variable expressivity related with the Lacrimo – auriculo –dento-digital syndrome (LADD), known as Levy-Hollister syndrome and characterized by aplasia, atresia or hypoplasia of the lacrimal and salivary systems, cup-shaped ears, hearing loss and dental and digital affections (8).

The fibroblast growth factor 10 (FGF10), a gene located in 5p13-p12 chromosome and encoding a protein of 508 amino acids, is involved in cellular morphogenesis, and essential in development process of lacrimal and salivary glands. It has been suggested in the recent literature that the mutation of this gene could be involved in the LADD and ALSG syndromes (1,8-11).

A few cases of aplasia of all major salivary glands has been reported in the literature, nevertheless incomplete aplasia or hypoplasia is described (1,2,4-6,12,13). Parotid is more usual affected but submandibular gland agenesis seems to be not common (14). According to our knowledge, in the Spanish population only two cases of salivary glands aplasia have been described, a bilateral aplasia of submandibular glands associated with a cleft lip palate (15) and one case of unilateral aplasia of parotid gland (16). Our case it’s the first of ALSG syndrome in the Spanish literature.

The correct function of oral tissue it’s possible by means of saliva, and the lack of function may cause dental caries, infections, xerostomia and difficulty of mastication and speech. Sialometry can be developed to demonstrate the diminished salivary flow (15). Alteration of lacrimal glands it’s related with irritable eyes symptoms, infections and even epiphora if the duct or puncta is involved (1). Nevertheless, the patients with ASLG syndrome usually don’t present any symptoms because the tear secretion is reduced but at the same time, balanced by the diminished drainage caused by alteration of the duct and/or puncta (17). This fact can be confirmed by a Schirimer test (12).

To have a high clinical suspicion is mandatory to diagnose ALSG (1). Important caries at early ages associated with dry mouth, lack of duct orifices and ocular symptoms should suggest an ALSG or related syndrome. Several etiologies have been described as responsible for dry mouth symptoms, drugs are the main cause (18). Differential diagnosis must include Sjörgen syndrome. In 66,4% patients the parotid gland may be enlarged and a minor salivary gland biopsy can confirm the diagnosis (1,18). Also it is suggested that in ASLG the symptoms are presented earlier (1). Serum test must be realized to reject a systemic syndrome (1,17,18).

Imaging tests are necessary to confirm the lack of formation of salivary glands and alteration of lacrimal system. Many techniques have been described such as US, scintigraphy, TC and RMI (1,2,4,7,15-23).

A mutation of FGF10 has been proposed as the responsible of the syndrome (1,8-11), but its low availability difficult the genetic confirmation (1,13,17).

The first line of treatment in ALSG syndrome it is early diagnosis to prevent future consequences, specially related with orodental alteration. Multiple saliva substitutes have been proposed such as glycerine and lemon, carboxymethhylcellulose, oil and water emulsions, lactoperoxidase, glucose oxidase, xylitol, and many others (2,4,5). Also pharmacological sialagogue such as pilocarpine has been proposed to improve the salivary function when a small part of it is still working (5,19). Acidic substitutes as glandosane should not be used in dentate patients (20).

Oral hygiene, high fluoride tooth paste and other agents like chewing gum can be useful. A regular visit to the dentist since the young ages it’s necessary to prevent rampant caries.

The management of the lacrimal alteration depends of the clinical findings and can include artificial tears and sometimes dacryocystectomy or occasional puncta (17,19). Although the tear drainage can be balanced by the alteration of duct, the secretion of the gland could be abnormal (17).

The patient was referred to our center at the age of 40. He had been consulting with the initial clinical suspicion of Sjörgen syndrome, which was refuted with a minor salivary glands biopsy. Despite the ophthalmologic surgery in the young ages and the absence of all salivary glands, no diagnosis of ALSG was proposed until he was visited by the department of Oral and Maxilofacial Surgery in A Coruña, Spain. A symptomatic treatment was indicated, but no cholinergic agent such as Pilocarpine was indicated because of the asthma medical history of the patient. The patient had no ocular symptoms, but regular ophthalmologic revisions were advised. Although no genetic confirmation of FGF10 mutation was available, our patient completes all the clinical findings of ALSG syndrome and according to our knowledge it represents the first case in the Spanish literature.

## Conclusions

ALSG syndrome could be caused by FGF10 mutation, but nowadays no genetic test is regularly available to confirm the alteration. Clinical suspicion remains the principal tool to diagnose the syndrome and it is essential to a correct management of ALSG. Orodental symptomatic treatment should be initiated at early ages to prevent caries, and ophthalmological review should carry out regularly.
